# Quantified fat fraction as biomarker assessing disease severity in rare Charcot–Marie–Tooth subtypes

**DOI:** 10.3389/fneur.2023.1334976

**Published:** 2024-01-29

**Authors:** Xingwen Sun, Xiaoxuan Liu, Qiang Zhao, Lihua Zhang, Huishu Yuan

**Affiliations:** ^1^Department of Radiology, Peking University Third Hospital, Beijing, China; ^2^Department of Neurology, Peking University Third Hospital, Beijing, China

**Keywords:** Charcot–Marie–Tooth disease, magnetic resonance imaging, muscle fat quantification, calf muscles, fat fraction

## Abstract

**Objective:**

Charcot–Marie–Tooth (CMT) disease is the most common inherited neuromuscular disorder. Multi-echo Dixon MRI technique is a highly sensitive method for quantifying muscle fatty infiltration, which may provide excellent value for the assessment of CMT. Due to the rareness of the disease, its use in CMT disease has been rarely evaluated, especially in subtypes.

**Methods:**

Thirty-four CMT1 patients, 25 CMT2 patients, and 10 healthy controls were recruited. All of the recruited CMT1 patients are CMT1A with PMP22 duplication. Among CMT2 patients, 7 patients are CMT2A with MFN2 mutation, and 7 patients have SORD mutations. Multi-echo Dixon MRI imaging was performed. The fat fractions (FFs) of 5 muscle compartments of the leg were measured at proximal, middle, and distal levels by two specialized musculoskeletal radiologists. Comparisons between CMT1, CMT2, and genetically defined subtypes were conducted.

**Results:**

A proximal-distal gradient (27.6 ± 15.9, 29.9 ± 19.7, and 40.5 ± 21.4, *p* = 0.015) with a peroneal predominance (*p* = 0.001) in fat distribution was observed in CMT1. Significant differences in the soleus muscle FFs at proximal (19.1 ± 14.7 vs. 34.8 ± 25.1, *p* = 0.034) and medial levels (23.5 ± 21 vs. 38.0 ± 25.6, *p* = 0.044) were observed between CMT1 and CMT2 patients. Between PMP2 duplication and MFN2 mutation group, a significant difference in the soleus muscle FF was also observed (23.5 ± 21.0 vs. 54.7 ± 20.2, *p* = 0.039). Prominent correlations of calf muscle FFs with functional scores were observed.

**Discussion:**

Multi-echo Dixon MRI imaging is a valuable tool for assessing disease severity in CMT. The difference in patterns of fatty infiltration of CMT subtypes is first reported, which could provide references when making targeted training plans.

## Introduction

Charcot–Marie–Tooth (CMT) disease is a pathologically and genetically heterogeneous motor and sensory neuropathy. It is the most common inherited neuromuscular disorder with a prevalence of 1 in 2500 (1). Initially, most CMT patients present with slowly progressive distal weakness and atrophy, which may manifest as foot drop and pes cavus. Sensory deficits are often present. With time, foot deformities may occur. The diagnosis of CMT is mainly made by history and physical examination, supported by electrodiagnostic testing. Based on the physical examination and electrophysiological findings, CMT is divided into multiple clinical types. CMT1 and CMT2 are the most common ones. CMT1 is characterized by slowed nerve conduction velocity (NCV) (<38 m/s) and evidence of demyelination. CMT2 is featured by a normal or subnormal NCV (>38 m/s) and evidence of axonal degeneration and regeneration (2, 3). Under many circumstances, genetic testing could be performed to confirm a subtype-specific diagnosis. Not all clinically diagnosed CMT cases could be associated with a certain gene mutation. A significant proportion of CMT2 cases could not be genetically characterized (4). For evaluation of the clinical severity and progression of the disease, multiple scoring systems based on comprehensive physical examination in combination with nerve conduction study remained the primary methods. Given that CMT is a slow progressive disease, the low sensitivity and inter-rater repeatability of physical examinations are the main problems encountered in clinical practice (4). Additionally, electrophysiological study usually cannot be conducted due to severely damaged nerve in the lower limb (5). Sensitive measures reflecting disease progression are in urgent need (4, 6). MRI has been increasingly adopted to monitor the disease progression in clinical practice (6–8). To date, semi-quantitative visual grading of the extent of fat infiltration on conventional MRI imaging is the most frequently used method (6, 8). Nevertheless, the visual grading method is highly observer-dependent and could not provide quantitative data with sufficient sensitivity. The MRI Dixon fat water separation technique, which is able to quantify tissue fat content on a 0%–100% fat-fraction scale (9), showed high reproducibility and responsiveness in monitoring intramuscular fat accumulation in CMT1A patients (10–12). Quantified muscle fatty infiltration has been reported to be well correlated with major clinical measures in CMT1A (5, 13). Studies using traditional semi-quantitative visual grading methods have reported different patterns of intramuscular fatty infiltration in CMT1 and CMT2. These studies are of limited case number with inconclusive results (5, 6, 11, 13–16). To the best of our knowledge, there has been no study describing the quantified fat infiltration in CMT2 using the MRI Dixon fat water separation technique. Moreover, targeted training of certain muscle groups was recommended by recently published treatment guidelines (17). Knowledge of the characteristic patterns of fatty infiltration in different CMT subtypes would be indispensable for making a tailored treatment plan.

Our study aimed to quantify and describe the fatty infiltration of lower limbs in a large cohort of CMT1 and CMT2 patients. Differences between CMT1 and CMT2 were analyzed. Exploratory comparisons between genetic subtypes were also performed. Additionally, we analyzed the correlations between the quantified intramuscular infiltration with clinical metrics within each group to assess the appropriateness of using quantified fatty infiltration as a parameter for the evaluation of disease severity.

## Materials and methods

### Study population

This study was reviewed and approved by the institutional ethics review board under the reference number 2019-005-002. Written informed consent was acquired from all recruited patients.

From December 2018 to January 2023, we recruited 27 CMT2 patients, 34 CMT1A patients, and 10 healthy controls. The healthy controls did not have any diagnosed neuromuscular disease and were negative in the neurological examination. The clinical diagnosis was based on symptoms, signs, family history (including assessment of family members when possible), and neurophysiology as previously described (3). Patients were suspected of CMT when one or more of the following clinical features were present: slowly progressive symptoms (including weakness, distal muscle atrophy, gait abnormalities, sensory deficits, or absent distal reflexes), foot deformities (pes cavus and hammertoes), and family history of pes cavus and CMT. For the suspected patients, nerve conduction study was performed to detect signs of demyelination (via slow conduction velocities, prolonged distal latencies, prolonged F response latencies, conduction block, and/or temporal dispersion) or/and axonal loss (via low amplitude nerve potentials). Patients were then classified into demyelination type (CMT1) or axonal type (CMT2) according to their family history, clinical features, and electrophysiological findings.

Genetic testing was performed for all patients to search for the causative gene mutations. Genetic characterization was only possible in a portion of CMT2 patients, as previously reported (18). In our study, all 34 CMT1 patients were CMT1A subtypes with PMP22 duplication, which is the most common causative mutation for CMT1. Seven CMT2 patients had SORD mutation, 7 CMT2 patients had mutations in MFN2, 2 CMT2 patients had mutations in MPZ, 1 CMT2 patient had a mutation in HSPB1, 1 CMT2 patients had a SARS mutation, and 1 CMT2 patient had a mutation in GDAP1. The other 8 CMT2 patients were negative in next-generation sequencing.

All CMT patients did not have any other neurological diseases that may interfere with the evaluation, including diabetic neuropathy, chronic inflammatory demyelinating polyneuropathy, amyotrophic lateral sclerosis, Guillain–Barré syndrome, radiculopathy caused by degenerative spine diseases, etc. All healthy controls and CMT patients do not have any safety-related contraindications for MRI exams.

## Clinical assessment

Demographic characteristics and medical records were documented on recruitment. The neurological examination was performed by an experienced neurologist (15 years of clinical experience) specializing in neuromuscular disease. The severity of CMT was evaluated using several functional scores. The CMT neuropathy score version 2(CMTNSv2) (19) is a composite score comprising symptoms, signs, and electrophysiologic study results. Its subscore, the CMT examination score version 2(CMTES) (19), is calculated by the sum of symptoms and signs. The CMTNS lower limb is the CMTNS score computed only for the lower limbs. The CMTNS lower limb motor is a subscore of the CMTNS lower limb comprising motor symptoms and strength of lower limbs.

### Electrophysiological study

Nerve conduction studies of median nerves, ulnar nerves, tibial nerves, and peroneal nerves were conducted in all patients on Keypoint G4 (9031A070, Alpinebiomed Aps, Denmark). The parameters were as follows: skin temperature 36°C, scanning speed 3 ms/D; sensitivity 0.2 mV/D; filter 5 Hz − 5 kHz; recording length 200 ms; internal trigger; repeat frequency 3 Hz; disposable self-sealing Ag/AgCl surface electrode; supine position; and room temperature 25°C.

### Imaging technique

All MR scans were prospectively performed using a SIEMENS 3T scanner (MAGNETOM Prisma, Siemens Healthineers). Patients were in a supine position in the scanner, and both legs were imaged. An 18-channel body coil was used. The coronal T1-weighted turbo spin echo (TSE) sequence and axial proton density (PD) TSE fat-suppressed (FS) sequences were obtained for anatomical references. An axial multiple gradient echo Dixon-based MRI sequence (Multi-echo Dixon; Siemens Healthineers) was recorded with subsequent reconstruction of fat and water-only images. FF, the proportion of water (W) to fat (F) signal as a percentage, was calculated using the following formula: FF = F/(W + F) × 100%. Axial fat fraction (FF) maps were generated by an automatic post-processing system. Scan parameters are listed in Supplementary Table S1.

### Image analyses

**Figure 1:**
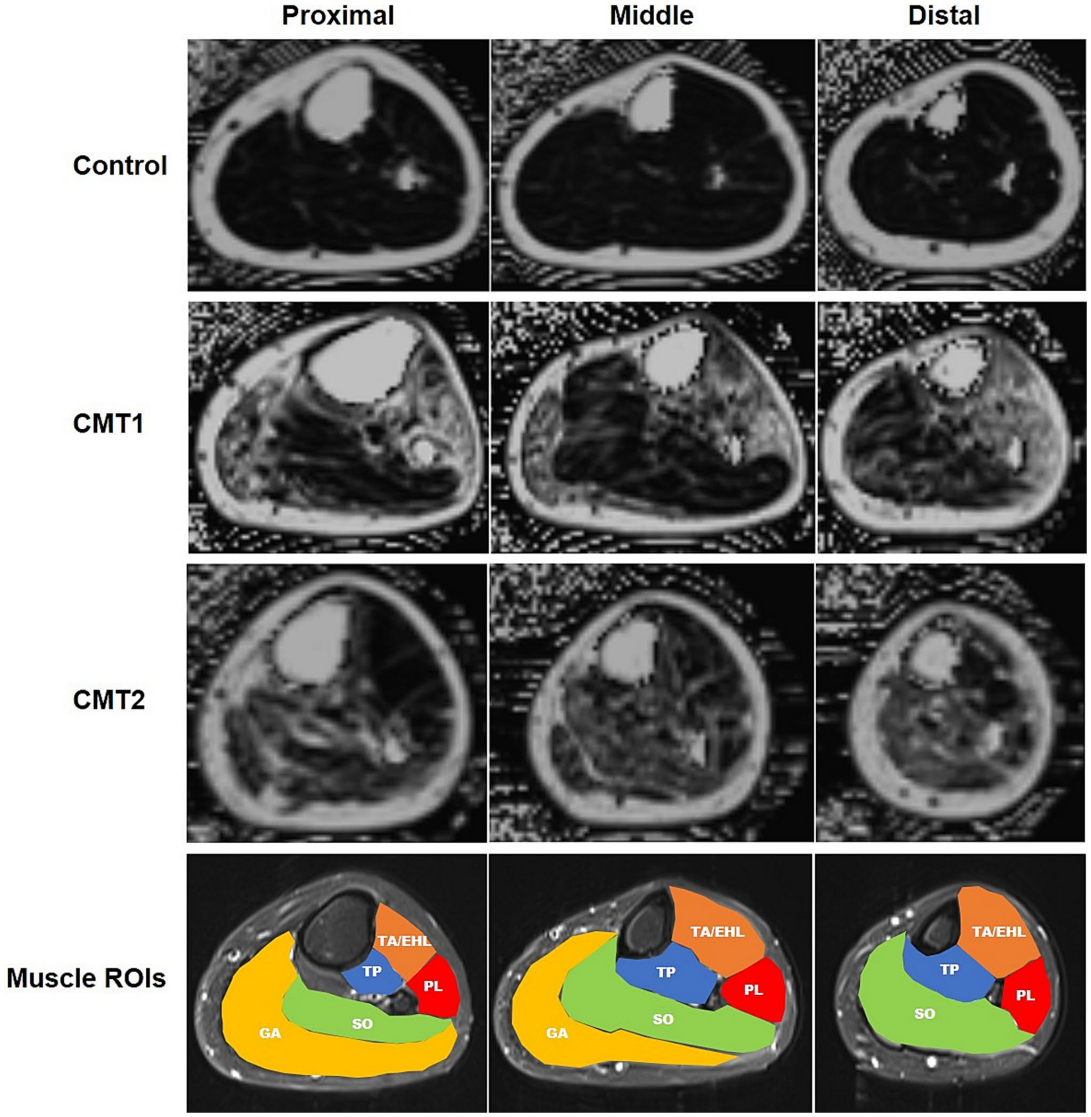
(Colored) Sample fat fraction maps and regions of interest of left lower limbs of CMT1, CMT2, and healthy controls at three levels. First three rows: fat-saturated proton density images at the proximal, medial, and distal levels in healthy control, CMT1, and CMT2 groups, respectively. Fourth row: regions of interest of leg muscles. TA/EHL, tibialis anterior/extensor hallucis longus; TP, tibialis posterior; PL, peroneus longus; SO, soleus; GA, gastrocnemius.

All image analyses were performed independently by two radiologists (XS and LZ with 4 and 16 years of experience in musculoskeletal imaging) on a picture archiving and communication system (Centricity^™^ PACS Radiology RA1000 Workstation) diagnostic workstation (GE Healthcare). Both readers were blinded to all related clinical information. The randomization process was performed by experienced an research coordinator. Leg muscles were manually segmented on the proximal, medial, and distal levels. One slice was selected on each level. Regions of interest (ROIs) masks were created on the axial PD TSE FS images to define the borders of the following muscles: the tibialis anterior and the extensor hallucis longus muscle (TA/EHL), the peroneus longus muscle (PL), the tibialis posterior muscle (TP), and the soleus muscle (SO) on all three levels (Figure 1). ROI mask of the gastrocnemius muscle (GA) was defined only on the proximal and medial levels due to its inconsistent demonstration on the distal level. ROI masks were then applied on co-registered FF maps to measure FFs. The mean fat fractions of both legs were calculated for further analysis.

### Statistical analysis

Statistical analysis was performed using SPSS version 26.0 (IBM). Analysis of variance (ANOVA) with *post hoc* analyses with Bonferroni correction was used for multiple comparisons of continuous clinical variables. Fisher’s exact test was used for comparisons of patients’ sex. Interobserver agreement of FF measurements was calculated using intraclass correlation coefficients (ICCs). The degree of agreement was interpreted using the following criteria: 0.8–1.0, excellent; 0.6–0.8, good; and <0.6, poor. FFs and ordinal clinical variables were presented as mean ± standard deviation (SD). We performed the Wilcoxon rank sum test to compare FFs and ordinal clinical variables given the non-gaussian distribution of the data. Kruskal–Wallis test with *post hoc* analyses with Bonferroni correction was conducted for multiple comparisons of FFs. *p* < 0.05 was considered significant for all tests. The Spearman rank correlation was used to analyze the correlations between metrics (*r*, Spearman *ρ*).

## Results

There was no significant difference in age, sex, disease duration, age of disease onset, and BMI between recruited CMT1 patients, CMT2 patients, and healthy controls (Table 1). Interobserver agreement of FFs was excellent for all measurements (Supplementary Table S2). Figure 1 illustrates examples of FF maps of healthy control, CMT1, and CMT2.

**Table 1:** Summary of demographic characteristics and results of clinical assessments.

	CMT1	CMT2	Control	*p*-values^†^
No. of patients	34	25	10	NA
Male:Female, *n*	24:10	16:9	5:5	0.377
Age, y	39.7 ± 13.6	32.7 ± 14.0	32.5 ± 8.7	0.186
BMI, kg/m^2^	24.8 ± 5.0	22.8 ± 3.3	24.8 ± 5.0	0.289
Age of disease onset	25.6 ± 16.0	24.9 ± 16.7	NA	0.593
Disease duration	14.1 ± 15.6	11.5 ± 8.6	NA	0.495
CMTNSv2	12.9 ± 3.5	9.4 ± 6.1	NA	0.012^*^
CMTES	6.9 ± 3.2	7.2 ± 4.2	NA	0.688
CMT lower limb	5.8 ± 2.7	5.8 ± 3.1	NA	0.628
CMT lower limb motor	3.4 ± 1.4	4.4 ± 1.4	NA	0.015^*^
Dorsiflexion	3.0 ± 1.8	2.5 ± 1.8	NA	0.272
Plantar flexion	4.0 ± 1.1	3.5 ± 1.4	NA	0.109

BMI, body mass index; CMT, Charcot–Marie–Tooth disease; CMTNSv2, Charcot–Marie–Tooth Neuropathy Score Version 2; CMTES, Charcot–Marie–Tooth Examination Score; NA, not applicable. ^†^ANOVA with post hoc analyses with Bonferroni correction was used for multiple comparisons of clinical variables, except sex ratio (Fisher’s exact test). ^*^p < 0.05.

### Comparisons of the total muscle FFs between the proximal, medial, and distal levels in CMT1, CMT2, and healthy controls

**Figure 2:**
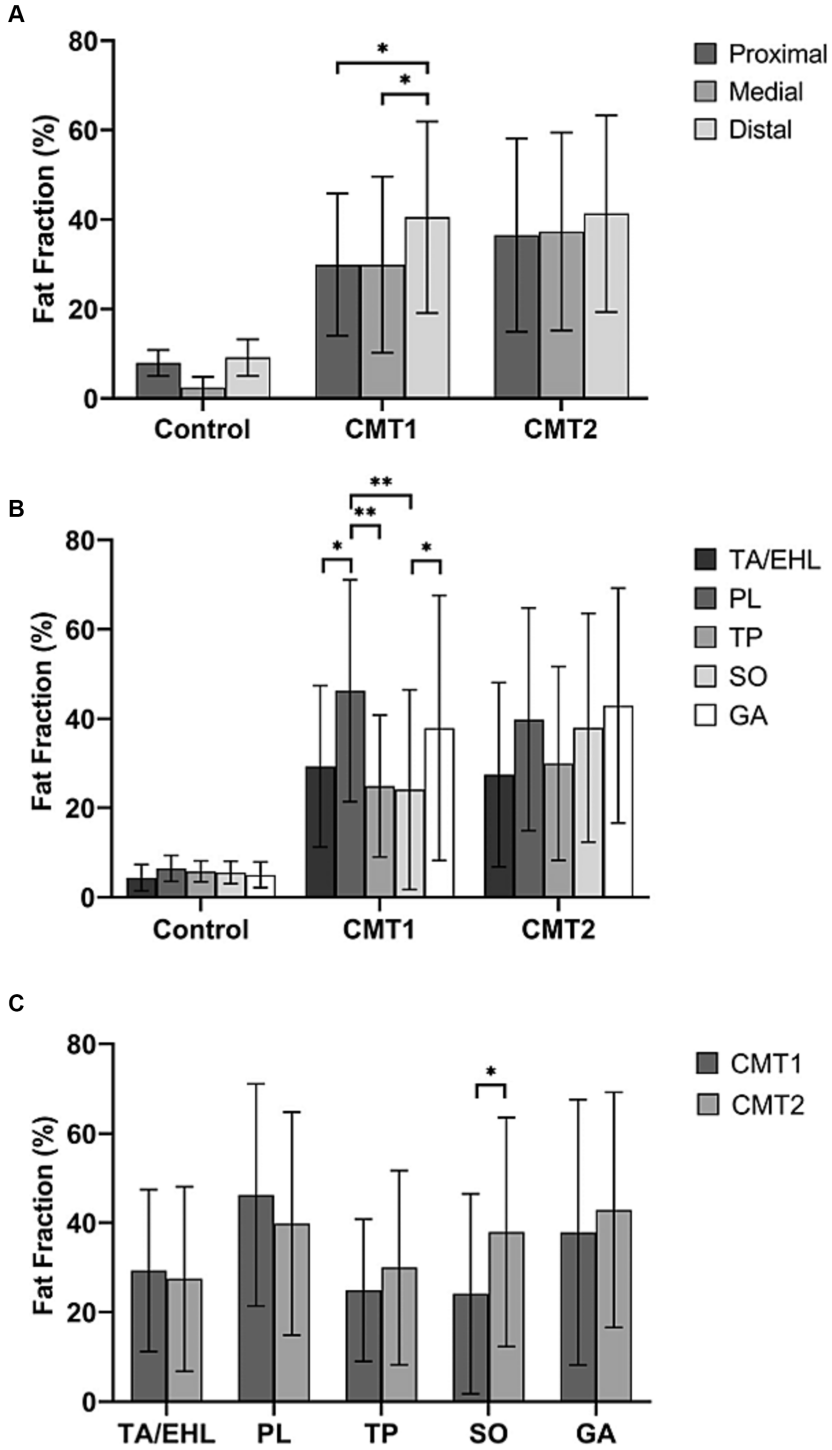
Total fat fractions of healthy controls, CMT1, and CMT2 patients at the proximal, medial, and distal levels and fat fractions of individual muscles. **(A)** Compared with healthy controls, CMT1 and CMT2 patients have significantly higher fat fractions at all three levels. The fat fraction of the distal level is significantly higher than the proximal and medial levels. Healthy controls and the CMT2 group did not show similar changes. **(B)** The fat fractions of individual muscles did not show significant differences in healthy controls and CMT2 patients. In CMT1A patients, the fat fraction of the peroneus longus muscle was significantly higher than the tibialis anterior/extensor hallucis longus muscle, tibialis posterior muscle, and soleus muscle. The FF of gastrocnemius is significantly higher than the soleus muscle. **(C)** Fat fraction of the soleus muscle is significantly higher in the CMT2 group than the CMT1 group.

Along the long axis of the leg, a significant increase from the proximal to the distal level was observed in the CMT1 group (Figure 2A). The FF of the distal level (40.5% ± 21.4%) was significantly higher than the proximal (27.6% ± 15.9%, *p* = 0.01) level and the medial level (29.9% ± 19.7%, *p* = 0.031). Healthy controls and the CMT2 group did not show similar changes. No significant difference was observed between different levels in healthy controls (7.9 ± 3.1%, 5.6 ± 2.5%, and 9.1 ± 4.3%, *p* = 0.078) and CMT2 group (36.5 ± 21.6, 37.3 ± 22.1, and 41.3% ± 22.0%, *p* = 0.699).

### Comparisons between individual muscle FFs at the medial level within the control, CMT1, and CMT2 groups

Figure 2B demonstrates comparisons of fat fractions of individual muscles at the medial level within each group. In the controls and the CMT2 group, no significant difference between individual muscles was observed (*p* = 0.118 and *p* = 0.222). In the CMT1A group, the FF of the PL (46.2% ± 24.9%) was significantly higher than that of the TA/EHL (29.3% ± 18.1%, *p* = 0.013), the TP (24.9% ± 15.9%, *p* = 0.001) and the SO (23.5% ± 21%, *p* < 0.001). The FF of the GA (37.9% ± 29.7%) was significantly higher than that of the SO (23.5 ± 21%, *p* = 0.036). No significant difference was found between TA/EHL, TP, and SO (*p* = 0.214).

### Comparisons of individual muscle FFs between the CMT1 group, CMT2 group, and healthy controls

**Figure 3:**
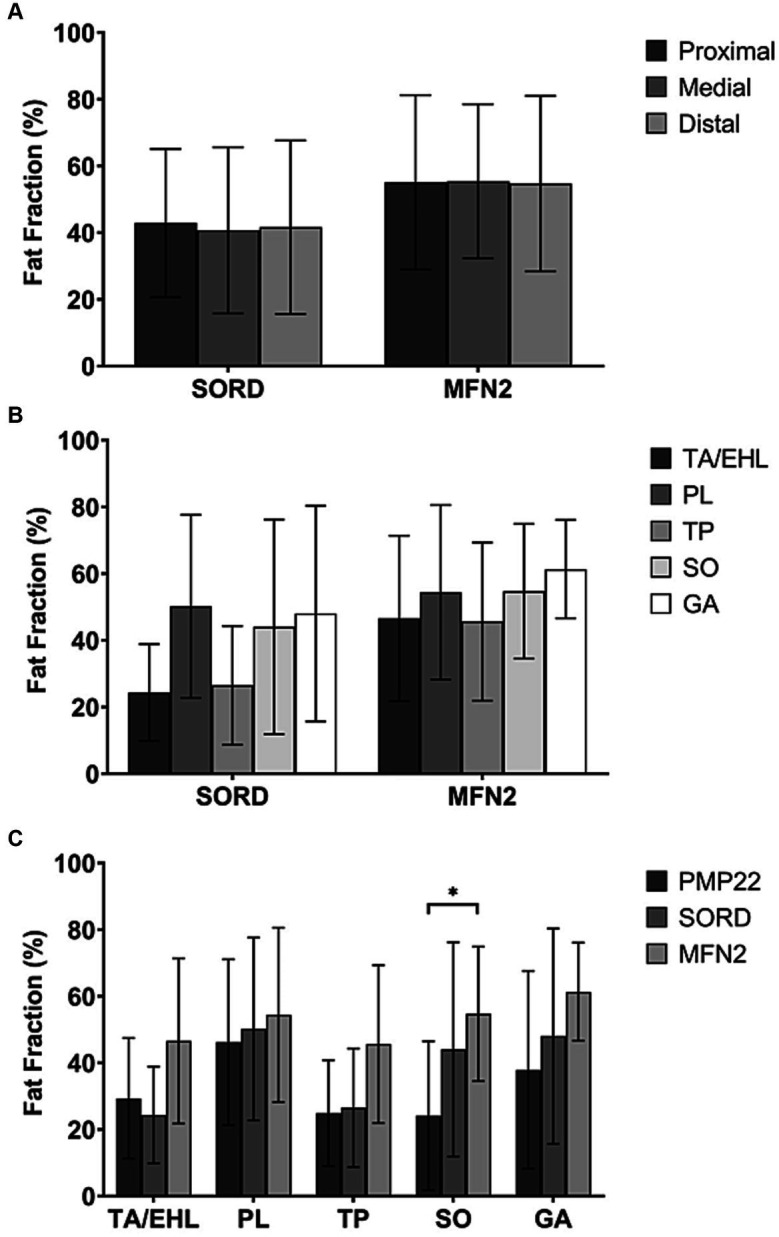
Total fat fractions of patients with different genetic subtypes at the proximal, medial, and distal levels and fat fractions of individual muscles. **(A)** No significant difference was observed between different levels in the SORD mutation group and the MFN2 mutation group. (We did not present the PMP22 duplication group here because it’s identical to the CMT1 group.) **(B)** The fat fractions of individual muscles did not show any significant difference in both SORD mutation group and the MFN2 mutation group. (We did not present the PMP22 duplication group here because it’s identical to the CMT1 group.) **(C)** The fat fraction of the soleus muscle in the MFN2 mutation group is significantly higher than that of the PMP22 duplication group.

Comparisons of the FFs between groups at all three levels were demonstrated in Table 2. Comparisons between CMT1 and CMT2 at the medial level were illustrated in Figure 3C. Compared with healthy controls, the FFs of all muscles in the CMT1 group and CMT2 group were all higher than that in healthy controls at all three levels. Comparing the CMT1 and CMT2 groups, the FF of the SO in the CMT2 group was significantly higher than in the CMT1 group at all three levels. The FF of the other muscles and the total FF in the CMT1 group and CMT2 group did not show significant differences at all three levels.

**Table 2:** Comparisons of muscle fat fractions between healthy volunteers, CMT1, and CMT2 at all three levels.

	Fat fraction (mean ± SD), %			*p*-value^†^		
	Controls	CMT1	CMT2	Control vs. CMT1	Control vs. CMT2	CMT1 vs. CMT2
**Proximal**						
TA/EHL	7.3 ± 3.3	25.5 ± 13.6	29.4 ± 21.8	<0.001^**^	<0.001^**^	0.891
PL	7.6 ± 2.9	33.6 ± 23.2	37.7 ± 24.3	<0.001^**^	<0.001^**^	0.636
TP	10.7 ± 5.1	28.0 ± 14.0	31.9 ± 19.6	<0.001^**^	<0.001^**^	0.630
SO	10.7 ± 5.1	19.1 ± 14.7	34.8 ± 25.1	0.002^**^	<0.001^**^	0.034^*^
GA	5.2 ± 2.3	30.7 ± 24.3	39.2 ± 24.8	<0.001^**^	<0.001^**^	0.134
Total	7.9 ± 3.1	27.6 ± 15.9	36.5 ± 21.6	<0.001^**^	<0.001^**^	0.149
**Middle**						
TA/EHL	4.4 ± 2.9	29.3 ± 18.1	27.4 ± 20.6	<0.001^**^	<0.001^**^	0.573
PL	6.5 ± 2.9	46.2 ± 24.9	39.8 ± 24.9	<0.001^**^	<0.001^**^	0.486
TP	5.8 ± 2.3	24.9 ± 15.9	30.0 ± 21.7	<0.001^**^	<0.001^**^	0.459
SO	5.5 ± 2.5	23.5 ± 21	38.0 ± 25.6	<0.001^**^	0.001^**^	0.044^*^
GA	5.0 ± 2.9	37.9 ± 29.7	42.9 ± 26.3	<0.001^**^	<0.001^**^	0.384
Total	5.6 ± 2.5	29.9 ± 19.7	37.3 ± 22.1	<0.001^**^	<0.001^**^	0.305
**Distal**						
TA/EHL	6.5 ± 2.4	42.0 ± 20.9	37.4 ± 20.4	<0.001^**^	<0.001^**^	0.482
PL	5.0 ± 2.0	42. 6 ± 18.2	41.0 ± 22.5	<0.001^**^	<0.001^**^	0.861
TP	8.5 ± 4.5	35.5 ± 22.5	37.5 ± 23.0	<0.001^**^	<0.001^**^	0.798
SO	9.2 ± 4.6	36.7 ± 24.0	47.0 ± 23.6	0.001^**^	<0.001^**^	0.088
Total	9.1 ± 4.3	40.5 ± 21.4	41.3 ± 22.0	<0.001^**^	<0.001^**^	0.811

CMT, Charcot–Marie–Tooth disease; CI, confidence interval; TA/EHL, tibialis anterior/extensor hallucis longus; PL, peroneus longus; TP, tibialis posterior; SO, soleus; GA, gastrocnemius. ^†^Kruskal–Wallis test with post hoc analyses with Bonferroni correction was conducted for multiple comparisons. ^*^p < 0.05 and ^**^p < 0.01.

### Comparisons of the proximal, medial, and distal levels within each genetically defined subtype

Similar to the entire CMT2 group, the CMT2 subtype with SORD mutation and the CMT2A subtype, characterized by a mutation in the MFN2 gene, did not show any significant difference between the proximal, medial, and distal levels (*p* = 0.932 and *p* = 0.926) (Figure 3A).

### Comparisons between individual muscle FFs at the medial level of each genetically defined subtype

Similar to the entire CMT2 group, no significant difference between individual muscle FFs was observed in both the SORD mutation group (*p* = 0.450) and the MFN2 mutation group (*p* = 0. 859) (Figure 3B).

### Comparisons of individual muscle FFs between genetically defined subtypes

All CMT1 cases recruited in our study were of CMT1A subtype with PMP22 duplication. Figure 3C showed comparisons of individual muscle FFs at the medial level between PMP22, SORD, and MFN2 mutation groups. MFN2 mutation group had a significantly higher FF of the SO (54.7% ± 20.2%) compared to the PMP22 duplication group (23.5% ± 21.0%, *p* = 0.039). There is no significant difference of the FF in SO between the PMP22 and the SORD mutation groups. The FFs of the TA/EHL (29.3 ± 18.1% vs. 24.3% ± 14.5% vs. 46.6% ± 24.8%, *p* = 0.205), PL (46.2 ± 24.9 vs. 50.2% ± 27.4% vs. 54.4% ± 26.2%, *p* = 0.724), TP (24.9% ± 15.9% vs. 26.5% ± 17.8% vs. 45.6% ± 23.7%, *p* = 0.146), and GA (37.9 ± 29.7% vs. 48.0% ± 32.3% vs. 61.4% ± 14.7%, *p* = 0.228) did not show significant difference between the three genetic heterogeneous groups.

### Correlations between muscle FFs and functional scores in CMT1 and CMT2 groups

In the CMT1 group, FFs of all calf muscles showed significant correlations with age, disease duration, CMTES, muscle force of dorsiflexion, and muscle force of plantar flexion. FFs of all calf muscles except PL were significantly correlated CMTNSv2 score. FF of SO was significantly correlated with both CMTNS lower limb and CMTNS lower limb motor scores. FF of PL was significantly correlated with CMTNS lower limb motor score (Table 3).

**Table 3:** *r* values^†^ of correlations between calf muscle fat fractions and clinical variables in CMT1 group.

	TA/EHL	PL	TP	SO	GA	Total
Age	**0.580** ^**^	**0.689** ^**^	**0.501** ^**^	**0.555** ^**^	**0.678** ^**^	**0.619** ^**^
Disease duration	**0.492** ^**^	**0.456** ^**^	**0.408** ^*^	**0.428** ^*^	**0.382** ^*^	**0.418** ^*^
**Functional scores**						
CMTNSv2	**0.390** ^*^	0.328	**0.407** ^*^	**0.535** ^**^	**0.448** ^**^	**0.514** ^**^
CMTES	**0.381** ^*^	**0.447** ^**^	**0.387** ^*^	**0.522** ^**^	**0.452** ^**^	**0.506** ^**^
CMTNS lower limb	0.241	0.328	0.331	**0.482** ^**^	0.318	**0.393** ^*^
CMTNS lower limb motor	0.305	**0.411** ^*^	0.306	**0.411** ^*^	0.289	**0.359** ^*^
**Muscle force**						
Dorsiflexion	**−0.473** ^**^	**−0.554** ^**^	**−0.400** ^*^	**−0.423** ^*^	**−0.423** ^*^	**−0.438** ^*^
Plantar flexion	**−0.450** ^**^	**−0.646** ^**^	**−0.491** ^**^	**−0.503** ^**^	**−0.453** ^**^	**−0.529** ^**^

CMTNSv2, Charcot–Marie–Tooth Neuropathy Score Version 2; CMTES, Charcot–Marie–Tooth Examination Score; TA/EHL, tibialis anterior/extensor hallucis longus; PL, peroneus longus; TP, tibialis posterior; SO, soleus; GA, gastrocnemius. ^†^The Spearman rank correlation was used to analyze the correlations between metrics. ^*^p < 0.05 and ^**^p < 0.01. The bold values are significant values.

In the CMT2 group, all calf muscles did not show any significant correlation with age and disease duration. The FF of TA/EHL was significantly correlated with all functional scores. Significant correlations were found between FFs of all muscles except GA and CMTNS lower limb motor. Regarding muscle force, TA/EHL, PL, TP, and total muscle FFs were significantly correlated with dorsiflexion strength. TA/EHL, PL, SO, GA, and total muscle FFs were significantly correlated with plantar flexion strength (Table 4).

**Table 4:** *r* values^†^ of correlations between calf muscle fat fractions and clinical variables in CMT2 group.

	TA/EHL	PL	TP	SO	GA	Total
Age	0.147	0.274	0.298	0.379	0.346	0.335
Disease duration	0.183	0.226	0.164	0.280	0.188	0.278
**Functional scores**						
CMTNSv2	**0.469** ^*^	0.303	0.363	0.258	0.293	0.332
CMTES	**0.433** ^*^	0.317	0.405	0.381	0.305	0.399
CMTNS lower limb	**0.495** ^*^	0.324	0.404	0.404	0.225	0.408
CMTNS lower limb motor	**0.659** ^**^	**0.514** ^*^	**0.467** ^*^	**0.498** ^*^	0.401	**0.493** ^*^
**Muscle force**						
Dorsiflexion	**−0.653** ^**^	**−0.586** ^**^	**−0.447** ^*^	−0.376	−0.260	**−0.460** ^*^
Plantar flexion	**−0.447** ^*^	**−0.530** ^*^	−0.357	**−0.528** ^*^	**−0.443** ^*^	**−0.510** ^*^

CMTNSv2, Charcot–Marie–Tooth Neuropathy Score Version 2; CMTES, Charcot–Marie–Tooth Examination Score; TA/EHL, tibialis anterior/extensor hallucis longus; PL, peroneus longus; TP, tibialis posterior; SO, soleus; GA, gastrocnemius. ^†^The Spearman rank correlation was used to analyze the correlations between metrics. ^*^p < 0.05 and ^**^p < 0.01. The bold values are significant values.

## Discussion

Evidence supporting the role of quantitative muscle MRI in evaluating disease severity and treatment efficacy is accumulating (4–6, 11, 13). Fat fraction showed a stronger correlation with strength and function when compared with clinical examination and myometric measures (11). Targeted training of certain muscle groups was recommended by the latest treatment guideline (17). Awareness of the difference in fatty infiltration of different CMT subtypes is critical when making individual exercise programs. Our study revealed the difference in the pattern of quantified fatty infiltration of lower limb muscles in CMT1, CMT2, and healthy controls. Exploratory analysis of the difference between genetic subtypes was also conducted. A statistically significant difference was detected between CMT2A (MFN2 mutation), the most common type of CMT2, and CMT1A (PMP22 duplication), the most common type of CMT1. Moreover, these findings may aid the differential diagnosis when the other findings are ambiguous or not readily available. Clinical correlation analysis in our study revealed prominent correlations of calf muscle FFs with clinical measurements, suggesting MRI with quantified fat fraction as an appropriate method to assess disease severity.

To date, the semi-quantitative visual grading method has been the mainly used method to assess muscular fat infiltration on MR in neuromuscular disorders (6, 10). In a few recent studies, the multi-echo Dixon technique enabling quantification of fat infiltration was reported to have a higher sensitivity than the semi-quantitative grading system (10–12). Before being applied in CMT disease, the multi-echo Dixon technique has been well validated in other neuromuscular diseases (11). The quantified fat fraction has been proposed as a valuable biomarker to monitor intramuscular fat accumulation with high responsiveness. Strong correlation with conventional functional measures suggested its validity (11, 13). Also, it showed excellent concordance with MR spectroscopy in intramuscular fat quantification (13, 20). The high sensitivity of the multi-echo Dixon technique may allow early identification and accurate assessment of the disease course, which is crucial for clinical management.

Several studies have investigated the pattern of fatty infiltration along the long axis in the lower limbs of CMT patients. In line with our study, multiple researchers have reported a proximal to distal gradient of fatty infiltration in the lower legs of CMT1 patients (5, 11, 13). For CMT2 patients, studies are scarce due to the rareness of the disease. In a recent study of 6 SORD neuropathy patients using semi-quantitative methods, a more prominent fat accumulation in the calf than thigh was reported. However, a quantified analysis of calf muscles was not performed (6). In another study of 5 CMT2F patients, the author described a more severe involvement of the lower thigh muscles than the upper thigh muscles. Similarly, this study is also limited by its semi-quantitative measures. No quantified data within the calf was available (14). Chung et al. (16) performed an analysis of the MRI features of 21 CMT2 patients. However, they did not analyze the distribution of fatty infiltration along the long axis of lower limbs. In our cohort of CMT2 patients, as well as in CMT2 subtypes with identical mutations, total FFs of different levels did not show any significant difference. This finding might be a result of a characteristic pattern of fatty infiltration in CMT2, but it could also occur when the recruited patients are at the late stage of the disease. Our cohort of CMT2 patients has 6 CMT2A subtypes with MFN2 mutation and 6 patients with SORD mutation. According to the current knowledge, MFN2 may present as early-onset CMT (21). Patients with MFN2 mutation may have highly variable manifestations. Heterogenous clinical findings were reported in family members sharing the same MFN2 molecular defect (22). This heterogeneity might be an explanation for the absence of the proximal-distal gradient and the difference between individual muscle groups. The natural course of patients with SORD mutation is not fully uncovered. Additionally, some of the patients with negative gene testing in our cohort also have a young age of disease onset, which may represent early-onset CMT2 subtypes that have not been genetically characterized. Further investigation of CMT2 at an early stage is warranted to give a comprehensive view of the development of fatty infiltration in the calf muscle.

A few studies have described distributions of fatty infiltration within individual muscles in CMT2 patients. However, the findings were rather variable. Gallardo et al. (23) described severe fatty replacement of all calf muscle compartments with relative preservation of the deep posterior one. In another study of 5 families of CMT type 2F patients, the pattern of involvement of the lower leg muscles was found to be predominantly T-type, which mainly involves the anterior compartments (14). A recent study performed by O’Donnell et al. (6) reported a prominent posterolateral involvement in 6 SORD mutation cases. These studies all used visual grading systems, which was not able to provide quantified data (10, 12). In our cohort of SORD mutation cases, although no statistical significance was observed, PL, SO, and GA showed a tendency to have a greater fat accumulation (Figure 3) than TA/EHL and TP. This finding is in accordance with O’Donnell et al. (6) and provided support to a posterolateral pattern of fat distribution in the CMT2 subtype with SORD mutation.

Among all the calf muscles, SO seems to be the one that may help clinicians distinguish between CMT1, CMT2, and even between genetically defined subtypes. Similar to our findings, a more predominant soleus involvement in the CMT2 group than in the CMT1A group was also identified by Chung et al. (16). CMT2A with MFN2 mutation is the most common subtype of CMT2, accounting for 10%–40% of all CMT2 cases. To date, there has been no study describing the pattern of fatty infiltration in CMT2A. Our study detected a significantly higher FFs of the soleus muscle in CMT2A compared with CMT1A with PMP22 duplication. This may indicate the difference in the natural disease course of genetically defined subtypes. MFN2 mutations lead to profound mitochondrial abnormalities, but the mechanism underlying the axonal pathology is still under investigation. In the PMP22 duplication subtype, excessive PMP22 in Schwann cells affects the processing and formation of compact myelin, which leads to dysmyelination. Further longitudinal studies with regular follow-up may reveal the cause of the difference in fatty infiltration.

Awareness of the difference in the pattern of fatty infiltration between CMT1, CMT2, and between subtypes is critical for clinical management because muscle fat infiltration is closely related to functional deficits and could predict deformity. Accurate assessment of the intramuscular fatty infiltration will allow tailored treatment planning, including rehabilitation therapy, customized external support, and surgical intervention (24–26). Additionally, the characterized fatty infiltration patterns could provide evidence supporting the diagnosis when ambiguity exists.

For CMT1A, the most common subtype of CMT, clinical correlations of fat infiltration are well established. Multiple large-scale clinical studies have provided solid evidence supporting the application of MRI quantified fat infiltration as a biomarker for disease progression assessment (5, 11, 13). Whereas for CMT2 or its subtypes, studies were limited, partially due to its rarity and genetic variety. As reported by O’Donnell et al. (6), there is a strong inverse correlation between ankle dorsiflexion strength and combined calf fat accumulation and atrophy for the 6 cases in their study. In our cohort of CMT2 patients, significant reverse correlations were also identified between calf muscle FFs and lower limb motor score as well as dorsiflexion strength. TA/EHL, the anterior muscle group, showed the highest *r* value. Due to the small case numbers of the SORD and MFN2 mutations, we were not able to validate the results within each individual genetic subtype, but current findings indicate promising results for the use of FF as a biomarker for disease progression monitoring in CMT2 as well.

Gender differences have also been reported in CMT1A patients. Female patients tend to have an earlier onset of symptoms than male patients and higher deterioration in quality of life (27). In our cohort, similar difference was not identified. Ten female cases may not be sufficient to detect the difference between sexes. Except for CMT1A, we were not able to recruit patients with other CMT1 subtypes, which limited the comparison between different genetic subtypes within the CMT1 group.

Using the MRI Dixon technique, we described the pattern of fatty infiltration of leg muscles in CMT1 and CMT2 patients. The quantified FFs showed significant differences between CMT1, CMT2, and genetically defined subtypes. Correlations with clinical variables provided more evidence for the value of FF in monitoring disease progression in both CMT1 and CMT2.

## Data availability statement

The original contributions presented in the study are included in the article/Supplementary material, further inquiries can be directed to the corresponding authors.

## Ethics statement

The studies involving humans were approved by Ethics Committee of Peking University Third Hospital. The studies were conducted in accordance with the local legislation and institutional requirements. Written informed consent for participation in this study was provided by the participants’ legal guardians/next of kin.

## Author contributions

XS: Conceptualization, Data curation, Formal analysis, Investigation, Methodology, Project administration, Software, Writing – original draft. XL: Conceptualization, Data curation, Funding acquisition, Investigation, Methodology, Resources, Writing – original draft, Writing – review & editing. QZ: Conceptualization, Data curation, Investigation, Writing – original draft, Writing – review & editing, Formal analysis, Project administration. LZ: Conceptualization, Data curation, Formal analysis, Project administration, Writing – review & editing, Funding acquisition, Methodology, Resources, Supervision, Validation. HY: Conceptualization, Data curation, Funding acquisition, Methodology, Project administration, Resources, Supervision, Writing – review & editing, Investigation, Writing – original draft.

## Figures and Tables

**Table:** 

ANOVA	Analysis of variance
BMI	Body mass index
CMT	Charcot–Marie–Tooth
CSA	Cross-sectional area
CMTES	Charcot–Marie–Tooth Examination Score
CMTNSv2	Charcot–Marie–Tooth Neuropathy Score Version 2
FF	Fat fraction
FS	Fat-suppressed
GA	Gastrocnemius
ICC	Intraclass correlation coefficient
MTR	Magnetization transfer ratio
PD	Proton density
PL	Peroneus longus
ROI	Region of interest
SD	Standard deviation
SO	Soleus
TA/EHL	Tibialis anterior/extensor hallucis longus
TP	Tibialis posterior
TSE	Turbo spine echo
