# Supplementary material for: Quantified fat fraction as biomarker assessing disease severity in rare Charcot–Marie–Tooth subtypes
Source: Front Neurol. 2024 Jan 29;14:1334976. doi: 10.3389/fneur.2023.1334976 (PMC10859536; doi:10.3389/fneur.2023.1334976)
Supplement: Supplementary file 1 [file Table_1.DOCX]

**Table S1. Scan parameters of imaging sequences.**

|  | **Multi-echo Dixon** | **T1-weighted TSE** | **PD TSE FS** |
| --- | --- | --- | --- |
| **Repetition time (ms)** | 9.07 | 550 | 4090 |
| **Echo time (ms)** | 1.26 | 8.9 | 63 |
| **Flip angle (°)** | 4 | 160 | 150 |
| **Number of averages** | 2 | 1 | 1 |
| **Reconstruction resolution (mm)** | 1.61 | 1.04 | 0.94 |
| **Matrix size** | 224*155 | 326*384 | 198*384 |
| **Field of view (mm)** | 314*359 | 399*399 | 247*360 |
| **Section thickness (mm)** | 4 | 5 | 5.5 |
| **Gap (mm)** | 0 | 0.75 | 0.83 |
| **Scan time(s)** | 100-121 | 55 | 66 |

**TSE**: turbo spin echo; **PD**: proton density; **FS**: fat saturated
