# Supplementary material for: Quantified fat fraction as biomarker assessing disease severity in rare Charcot–Marie–Tooth subtypes
Source: Front Neurol. 2024 Jan 29;14:1334976. doi: 10.3389/fneur.2023.1334976 (PMC10859536; doi:10.3389/fneur.2023.1334976)
Supplement: Supplementary file 2 [file Table_2.DOCX]

**Table S2. Interobserver agreement of muscle fat quantification.**

|  |  | **Interobserver agreement** | | | | | |
| --- | --- | --- | --- | --- | --- | --- | --- |
|  |  | **Proximal** | | **Medial** | | **Distal** | |
|  |  | **ICC** | **95%CI** | **ICC** | **95%CI** | **ICC** | **95%CI** |
| **Tibialis anterior/**  **Extensor hallucis longus** | **Right** | 0.984 | 0.967-0.993 | 0.988 | 0.976-0.994 | 0.991 | 0.979-0.996 |
|  | **Left** | 0.987 | 0.973-0.994 | 0.990 | 0.985-0.995 | 0.991 | 0.980-0.996 |
| **Peroneus longus** | **Right** | 0.996 | 0.990-0.998 | 0.993 | 0.987-0.997 | 0.994 | 0.987-0.997 |
|  | **Left** | 0.995 | 0.989-0.998 | 0.994 | 0.987-0.997 | 0.985 | 0.968-0.993 |
| **Tibialis posterior** | **Right** | 0.984 | 0.965-0.993 | 0.986 | 0.970-0.993 | 0.992 | 0.983-0.996 |
|  | **Left** | 0.988 | 0.976-0.995 | 0.990 | 0.980-0.995 | 0.992 | 0.984-0.996 |
| **Soleus** | **Right** | 0.993 | 0.986-0.997 | 0.993 | 0.985-0.997 | 0.991 | 0.981-0.996 |
|  | **Left** | 0.991 | 0.982-0.996 | 0.994 | 0.988-0.997 | 0.995 | 0.989-0.998 |
| **Gastrocnemius** | **Right** | 0.996 | 0.992-0.998 | 0.996 | 0.991-0.998 | NA | NA |
|  | **Left** | 0.995 | 0.989-0.998 | 0.995 | 0.990-0.998 | NA | NA |
| **Total** | **Right** | 0.989 | 0.976-0.995 | 0.988 | 0.976-0.994 | 0.989 | 0.976-0.995 |
|  | **Left** | 0.990 | 0.979-0.995 | 0.992 | 0.984-0.996 | 0.989 | 0.978-0.995 |

**ICC:** intraclass correlation coefficient; **CI**: confidence interval
